# Impact of axillary node-positivity and surgical resection margins on survival of women treated for breast cancer in Ibadan, Nigeria

**DOI:** 10.3332/ecancer.2020.1084

**Published:** 2020-08-05

**Authors:** Omobolaji O Ayandipo, Gabriel O Ogun, Olalekan J Adepoju, Ebenezer O Fatunla, Adefemi O Afolabi, Peter C Osuala, Temidayo O Ogundiran

**Affiliations:** 1 Department of Surgery, College of Medicine, University of Ibadan and University College Hospital, Ibadan, Nigeria; 2 Department of Pathology, College of Medicine, University of Ibadan and University College Hospital, Ibadan, Nigeria; 3 Department of Surgery, University College Hospital, Ibadan, Nigeria; 4 Department of Pathology, University College Hospital, Ibadan, Nigeria; a https://orcid.org/0000-0002-6806-8015; b https://orcid.org/0000-0003-0786-3311

**Keywords:** survival, breast cancer, Ibadan, axillary nodes, resection margins

## Abstract

**Introduction:**

Oncologic surgical extirpation, the mainstay of loco-regional disease control in breast cancer, is aimed at achieving negative margins and lymph node clearance. Even though axillary lymph nodal metastasis is a critical index of prognostication, establishing the impact of lymph node ratio (LNR) and adequate surgical margins on disease-specific survivorship would be key to achieving longer survival. This study examines the prognostic role of pN (lymph nodes positive for malignancy), LNR and resection margin on breast cancer survival in a tertiary hospital in Ibadan, Nigeria.

**Methods:**

We conducted a longitudinal cohort study of 225 patients with breast carcinoma, documented clinico-pathologic parameters and 5-year follow up outcomes – distant metastasis and survival. Chi-square test and logistic regression analysis were used to evaluate the interaction of resection margin and proportion of metastatic lymph nodes with patients’ survival. The receiver operating characteristic curve was plotted to determine the proportion of metastatic lymph nodes which predicted survival. The survival analysis was done using Kaplan–Meier method.

**Results:**

Sixty (26.7%) patients of the patients had positive resection margins, with the most common immuno-histochemical type being Lumina A. 110 (49%) patients had more than 10 axillary lymph nodes harvested. The mean age was 48.6 ± 11.8 years. Tumour size (p = 0.018), histological type (*p* = 0.015), grade (*p* = 0.006), resection margin (*p* = 0.023), number of harvested nodes (*p* < 0.01), number of metastatic nodes (*p* < 0.001) and loco-regional recurrence (*p* < 0.01) are associated with survival. The overall 5-year survival was 65.3%.

**Conclusion:**

Unfavourable survival outcomes following breast cancer treatment is multifactorial, including the challenges faced in the multimodal treatment protocol received by our patients.

## Introduction

Female breast cancer, the most commonly diagnosed cancer in women and a leading cause of female cancer mortality, continues to witness a rising age-standardized incidence rate [1–3]. The findings from an Ibadan population-based cancer registry in 2012 showed an incidence rate of 52.0 per 100,000 [3]. They are typically late stage, high grade and hormone receptor negative, and these factors represent an aggressive phenotype [4, 5].

Late stage at presentation has been associated with increased risk of mortality, especially in Low- and middle-income countries where social factors play a pivotal role in timing of presentation as well as acceptance of orthodox treatment, including breast-conserving surgery and mastectomy [6–8]. While surgical extirpation continues to be the mainstay of loco-regional disease control by achieving negative margins and lymph node clearance, it is well documented that the nodal status at diagnosis significantly affects overall survival, with axillary lymph nodal metastasis being a critical index of prognostication [9–11]. Furthermore, lymph node ratio (LNR), defined as the proportion of harvested lymph nodes positive for malignancy to total resected nodes [12, 13], has been implicated as a key prognostic factor. High LNR has not only been associated with unfavourable survival outcomes, its prognostic effect is found to be superior to total number of nodes [11]. Although the goal of surgery in breast cancer is to extirpate both macroscopic and microscopic tumour presence, the presence of a positive resection margin necessitates adjuvant radiotherapy with or without chemotherapy [14–16]. The late stage of disease in our environment makes modified radical mastectomy more favourable to a breast conserving surgery since in these cases, aggressive local therapy ensures adequate surgical margins and can determine disease-specific survival [17]. It is hoped that the utilisation of such multimodal approach to breast cancer care would keep the 5-year survival rate in sub-Saharan Africa on the upward trend [18–20]. Therefore, an intensive multimodal approach to breast cancer care is key to achieving longer disease-free survival and overall survival times [10, 11].

This study examines the prognostic role of pN (lymph nodes positive for malignancy), LNR and resection margin on breast cancer survival in a tertiary health institution in Ibadan, Nigeria.

## Methods

The study involved a longitudinal cohort of patients with breast carcinoma who underwent modified radical mastectomy or breast conserving surgery with axillary clearance at the Oncological Surgery Division of the University College Hospital, Ibadan, between December 2009 and December 2014. Ethical approval for this study was obtained from the Ministry of Health, Oyo State, Nigeria.

Preoperative tissue diagnosis was obtained by core needle biopsy and disease staging was done with chest radiography, abdominal sonography and bone scintigraphy.

The breast and axillary specimens were examined by dedicated breast pathologists, specifically for histological type and grade, axillary nodal metastasis and resection margin (defined as negative or positive). While the aim of surgical extirpation is to achieve complete cancer removal with at least 1cm cuff of grossly normal tissue, microscopic presence of tumour at the edges of excised specimen or axillary tail as well as underlying pectoral fascia classifies the surgical resection margin as positive [17]. Immunohistochemistry was done to determine receptor status. Adjuvant therapy consisted of chemotherapy, radiation treatment, endocrine therapy, immunotherapy or a combination of these.

The patients were followed up via clinic visits for a maximum period of 5 years following the completion of treatment, and contact tracing was done through phone calls, especially when the patients failed to turn up for scheduled appointments. Outcomes of interest included distant metastasis and survival. Statistical analysis was performed using Statistical Package for Social Sciences software (version 22; SPSS Inc. Chicago, IL). Descriptive statistics was used to examine the demographic and clinico-pathological profile of the patients. Chi-square test and logistic regression analysis were used to evaluate the interaction of resection margin and proportion of metastatic lymph nodes with patients’ survival. The receiver operating characteristic (ROC) curve was plotted to determine the proportion of metastatic lymph nodes which predicted survival. The survival analysis was done using Kaplan–Meier method.

The statistical significance was set at *p* < 0.05.

## Results

A total of 225 patients were recruited for the study. Table 1 shows their biodata. The mean age was 48.6 ± 11.8 years. Nearly half of the patients were civil servants.

As shown in Table 1, the histopathologic grossing average tumour size was 6 cm from with two-third of patients having tumour sizes above 5 cm. Nine of every ten participants had invasive carcinoma, not otherwise specified (NOS). The commonest tumour grade was the intermediate variety (49.8%; *n* = 112), followed by low-grade breast cancers. Almost all the participants underwent a modified radical mastectomy (99%; *n* = 222). Three patients who had pre-operative histology of malignant phyllodes tumour had clinically positive axillae and had dissection done.

The resection margin was free in 72% of patients with the most common immunohistochemical type being Lumina A, followed by the triple negative variety.

Majority of patients (49%; *n* = 110) had more than 10 axillary lymph nodes harvested with a mean 11 nodes (Table 2).

Nearly half of the patients (49.8%; *n* = 112) had an adjuvant chemotherapy alone whereas as high as 47.6% had no adjuvant care. As high as 28% mortality was recorded as shown in Table 2.

### Survival

Tumour size (*p* = 0.018), histological type (*p* = 0.015), grade (*p* = 0.006), resection margin (*p* = 0.023), number of harvested nodes (*p* < 0.01), number of metastatic nodes (*p* < 0.001) and loco-regional recurrence (*p* < 0.01) are associated with survival (Table 3).

Table 4 shows adjusted and unadjusted hazard rates using the Cox regression model. Without adjusting for confounders, factors associated with survival were tumour size, tumour grade, resection margin, clinical evidence of locoregional recurrence, number of harvested nodes and metastatic nodes. Patients with an involved resection margin had 72% more hazard rate than those with free resection margin (HR = 1.72, *p* = 0.037) while those with a recurrent cancer had three times the hazard rate of those with no breast cancer recurrence (HR = 3.21, *p* < 0.001).

**Figure 1.**
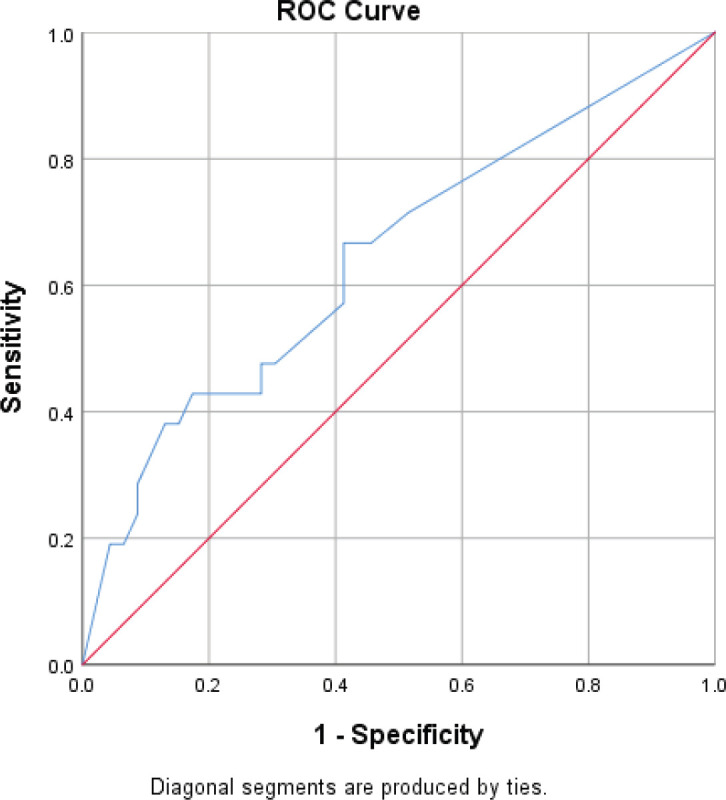
ROC curve for predicting survival using proportion of metastatic lymph nodes.

Patients with 1–3 metastatic nodes had 61% hazard rate from breast cancer (HR = 0.61, *p* = 0.235). This risk is 3.6 times higher in those with 4–9 metastatic nodes (HR = 2.19, *p* = 0.013), and 52 times higher in those with 10 or more metastatic nodes (HR = 31.62, *p* < 0.001). The above risk becomes higher when the hazard rate is adjusted. In patients who died, the proportion of metastatic lymph nodes was 33%, whereas 13% of metastatic lymph nodes were found in patients who survived (*p* = 0.001). With a sensitivity and specificity of 62% and 59% respectively, the cut-off proportion of metastatic lymph node that predicted mortality was 26% (Figure 1).

**Figure 2.**
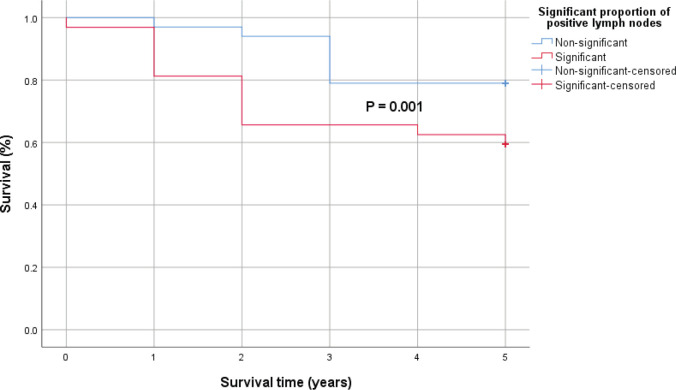
Kaplan–Meier survival curve comparing the 5-year survival of patients with significant proportion of positive lymph nodes (red) and those without (blue) with 0.001 *p*-value by log-rank test.

**Figure 3.**
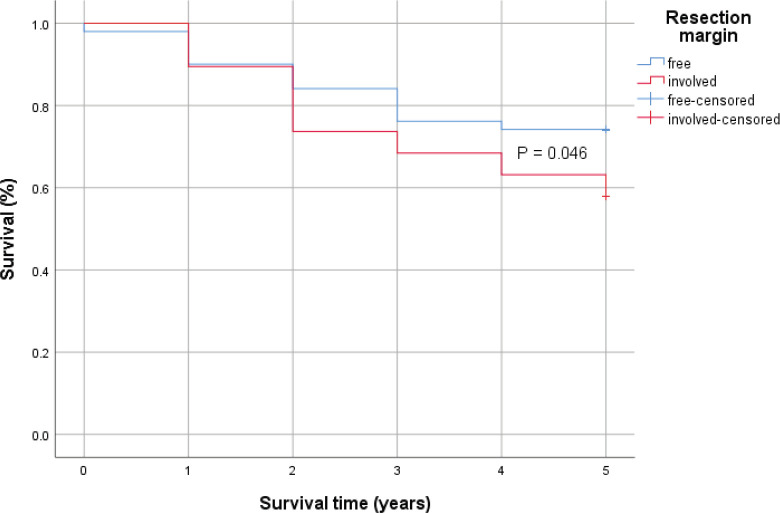
Kaplan–Meier survival curve comparing the 5-year survival of patients with positive resection margin (Red) and those without (Blue) with 0.046 *p*-value by log-rank test.

Patients with significantly higher proportion of positive lymph nodes (≥26%) recorded a statistically significant lower 5-year survival rates compared to those with lower positive lymph nodes (<26%). However, the difference in the 5-year survival outcomes with respect to positivity of resection margins was not statistically significant using the log-rank test (Figures 2 and 3).

## Discussion

The clinico-epidemiologic characteristics of our cohort tallies with findings from other series in the sub-region in terms of mean age (46.8–49) [21–23], occupation [21, 23] and tumour sidedness. There is no specific pattern of predilection for tumour sidedness – 53.8% (121) of cancers involved the left breast as compared to a previous study from our division in which 52.2% of tumours were on the right [23]. The histologic type of 9 out of 10 breast cancer cases were invasive ductal carcinoma (IDC). Other investigators found similar figures: Kim *et al* [10] – 88.5% and Tonellotto *et al* [11] – 87%. However, the proportion of IDC obtained in a review carried out a decade earlier in the same centre was 70.3% [23]. About two-thirds (65.5%) of the tumours were either intermediate or high Scarff–Bloom–Richardson grade, consistent with regional epidemiology [22–24].This study highlights the molecular classification of breast carcinoma immunohistochemical subtypes into Lumina A (ER+/PR+), Lumina B (ER+/PR+/HER-2+), Basal-like (triple negative) and HER-2 enriched tumours, unlike most local studies in literature which provide hormone receptor stratification in isolated patterns, presenting an unorganised reference for comparison.

The overall median 5-year survival was 65.3%, as compared with 79% reported in some high-income countries (HIC) [25]. This disparity in survival outcome figures with HICs, despite our patients also receiving multimodal and integrated breast cancer care, could be as a result of a high proportion of premenopausal patients who present with advanced disease with unfavourable tumour grade and immunohistochemical pattern [14, 22]. Also, inability to complete standard adjuvant treatment regimen by some patients due to limited infrastructure and lack of funds [23] contributes to this outcome, as health care funding is mainly out of pocket. For example, the patients in our cohort do not routinely have granulocyte-monocyte colony-stimulating factor (GM-CSF) prophylaxis as per National Comprehensive Cancer Network protocol [26] but are given therapeutically and sometimes have to postpone some chemotherapy courses as a result of cost when they have haematologic chemotoxicity. Even after down staging with neoadjuvant chemotherapy and undergoing mastectomy, availability of external beam radiotherapy is limited in the country [27].

Resection margins were histologically free of cancer in almost three-quarters of the mastectomy specimens (72.4%). This is comparable to 73.5% found by Kim *et al* [10] Tumour involvement ranged from edges of excised specimen and axillary tail to underlying pectoral fascia. We did not perform a routine re-operation for microscopic residual cancer, rather adjuvant chemoradiation therapy was commonly utilised.

An average of 11 axillary nodes were harvested per patient, which is lower than 26 (range: 10–61) found in South Korean women [10], 19 (range: 6–77) documented by Tonellotto [11] and 15 (range: 2–31) reported by Abass *et al* [28] in Sudan, who also submitted that axillary lymph node dissection is considered efficacious if >10 nodes are harvested [29]. The lower yield in our series may be attributable to the fact that more than 50% of our patients had neoadjuvant chemotherapy which has been shown to reduce the number of lymph nodes retrieved in axillary dissection specimen [30]. The axillary dissection procedures were done by experienced breast surgeons and specimen grossing conducted by dedicated breast pathologists, which minimises the likelihood of inadequate clearance or retrieval. Based on the staging system of the American Joint Committee on Cancer and The Union for International Cancer Control (AJCC/UICC) into pN1 (1‒3 positive lymph nodes), pN2 (4‒9 positive lymph nodes) and pN3 (≥10 positive lymph nodes), 47 patients were classified as pN1 (21%), 63 (28%) as pN2 and 12 (5.3%) as pN3 according to the number of positive lymph nodes.

While routine axillary node dissection is not recommended in phyllodes tumour as nodal involvement is rare [31], many authors support nodal clearance in cases of palpable axillary nodes [32, 33], even though only 1.1%–3.8% of clinically evident nodes have been reported to be pathologically positive [31]. Palpable nodes in three patients with phyllodes tumour in our series necessitated clearance.

Tumour size, tumour grade, resection margin, evidence of recurrence, harvested nodes and metastatic nodes were associated with survival. Hazard rates in the Cox regression analysis, however, showed that after adjusting for confounders, patients with cancer recurrence were about four times less likely to survive till the end of the follow up period (95% CI, 1.92–5.36; *p* < 0.001).

The trade-off plot of the ROC curve present classifiers with a sensitivity and specificity of 62% and 59%, respectively, the cut-off proportion of histologically positive lymph node predicting disease-specific 5-year mortality being 26%.

In cases with significantly higher proportion of malignant lymph nodes (≥26%), there was a statistically significantly lower 5-year survival rates compared to those with lower positive lymph nodes (<26%). Even though patients with microscopic residual tumour in resection margins had 72% more hazard rate than those with negative resection margin (HR = 1.72, *p* = 0.037) in a time-to-event analysis, the difference in the 5-year survival outcomes with respect to positivity of resection margins was not statistically significant using the log-rank test (*p*= 0.046).

## Conclusion

The clinico-epidemiology characteristics of our patients are similar to that seen worldwide; a high positive lymph node ratio is associated with poorer survival rates. Tumour recurrence is also associated with poor survival and is seen commonly with positive resection margins. However, the 5-year survival outcome was unfavourable. This is attributed to limited infrastructure, e.g., radiotherapy service, lack of funds and late clinico-pathologic presentation by patients.

## Limitations

Challenges in delivery of complete multimodal treatment regimen to our patients, including unbearable costs, suboptimal access to radiation therapy and poor uptake of immunotherapy.

## Conflicts of interest

The authors declare that they have no competing interests.

## Funding

None.

## Figures and Tables

**Table 1.** Biodata and Clinico-pathologic profile.

	Frequency *n* = 225	Percentage %
Age **(48.6 ± 11.8)**		
< 35 years	27	12.0
35–44 years	64	28.4
45–54 years	65	28.9
55–64 years	45	20.0
≥ 65 years	24	10.7
Occupation		
Civil servant	109	48.4
Trader	101	44 .9
Farmer	3	1.3
Others	12	5.3
Breast cancer sidedness		
Right	104	46.2
Left	121	53.8
Tumour size (cm) [6 (4)]		
*T_0_* (0cm)	3	1.3
*T_1_* (≤2cm)	12	5.3
*T_2_* ( 2.01–5cm)	63	28.0
*T_3_* (>5cm)	147	65.3
Histological type		
Invasive CA NOS	203	90.2
Invasive lobular CA	13	5.8
Phyllodes tumour	3	1.3
Ductal carcinoma in situ (DCIS)	6	2.7
Tumour grade		
Low grade	39	17.3
Intermediate grade	112	49.8
High grade	36	16.0
Not stated	38	16.9
Resection margin		
Free	163	72.4
Involved	60	26.7
Unknown	2	0.9
Immunohistochemistry		
Luminal A	69	30.7
Luminal B	6	2.7
Her-2-enriched	2	9.3
Triple negative	35	15.6
Not stated	94	41.7
Nature of surgery		
MRM	222	98.7
Quadrantectomy + axillary clearance	3	1.3

**Table 2.** Axillary lymph nodal status, adjuvant treatment and outcomes.

	Frequency (n = 225)	Percentage (%)
Harvested nodes [8 (6)]		
0	12	5.3
1–3	31	13.8
4–9	72	32.0
≥10	110	48.9
Metastatic Nodes [1 (4)]		
0	103	45.8
pN1: 1–3	47	20.9
pN2: 4–9	63	28.0
pN3: ≥10	12	5.3
Adjuvant therapy		
None	107	47.6
Chemotherapy	112	49.8
Chemo + RTH	3	1.3
Tamoxifen	3	1.3
5-year survival status		
Alive	147	65.3
Dead	63	28.0
Unknown	15	6.7

**Table 3.** Factors associated with survival.

	Alive	Dead	Chi-square (*p*-value)
Tumour size (cm)			
0	3 (100%)	0	10.09 (0.018)
≤2	9 (75%)	3 (25%)	
2.01–5	48 (84.2%)	9 (15.8%)	
>5	87 (63%)	51 (37%)	
Histological type			
Invasive CA NOS	125 (66.5%)	63 (33.35%)	10.53 (0.015)
Invasive lobular CA	13 (100%)	0	
Phyllodes tumour	3 (100%)	0	
Ductal carcinoma in situ (DCIS)	6 (100%)	0	
Tumour grade			
Low grade	21 (53.8%)	18 (46.2%)	10.34 (0.006)
Intermediate grade	70 (68%)	33 (32%)	
High grade	27 (90%)	3 (10%)	
Resection margin			
Free	112 (74.2%)	39 (25.8%)	5.19 (0.023)
Involved	33 (57.9%)	24 (42.1%)	
Evidence of recurrence			
Yes	138 (78%)	39 (22%)	34.04 (<0.01)
No	9 (27.3%)	24 (72.7%)	
Harvested nodes			
0	9 (100%)	0	27.98 (<0.01)
1–3	28 (90.3%)	3 (9.7%)	
4–9	33 (47.8%)	36 (52.2%)	
≥10	77 (76.2%)	24 (23.8%)	
Metastatic nodes			
0	76 (80.9%)	18 (19.1%)	21.27 (<0.001)
1–3	35 (74.5%)	12 (25.5%)	
4–9	33 (57.9%)	24 (42.1%)	
≥10	3 (25%)	9 (75%)	
Metastatic nodes			
No positive node	76 (80.9%)	18 (19.1%)	9.54 (0.002)
1 or more positive node(s)	71 (61.2%)	45 (38.8%)	

**Table 4.** Cox regression model showing adjusted and unadjusted hazard rates.

	Unadjusted HR (95% CI)		*p*-value	Adjusted HR (95% CI)	*p*-value
Tumour size (cm)					
≤2	0.29 (0.09–0.92)	0.036		NA	
2.01–5	0.60 (0.29–1.24)	0.170		1.06 (0.43–2.60)	0.894
>5	1			1	
Tumour grade					
Low grade	1			1	
Intermediate grade	0.78 (0.44–1.39)	0.401		0.39 (0.17–0.91)	0.028
High grade	0.21 (0.06–0.72)	0.013		0.12 (0.03–0.45)	0.002
Resection margin					
Free	1				
Involved	1.72 (1.03–2.87)	0.037		1.41 (0.70–2.84)	0.334
Evidence of recurrence					
Yes	3.21 (1.92–5.36)	<0.001		3.69 (1.51–9.02)	0.004
No	1				
Harvested nodes					
1–3	0.19 (0.06–0.63)	0.006		0.43 (0.08–2.20)	0.308
4–9	0.40 (0.23–0.68)	0.001		2.16 (0.87–5.39)	0.098
≥10	1			1	
Metastatic nodes					
0	1			1	
1–3	0.61 (0.27–1.37)	0.235		1.46 (0.49–4.37)	0.496
4–9	2.19 (1.18–4.04)	0.013		3.61 (1.52–8.60)	0.004
≥10	31.62 (11.30–8.47)	< 0.001		48.25 (10.17–228.89)	<0.001
